# Supplementary material for: Symptoms of Infarction in Women: Is There a Real Difference Compared to Men? A Systematic Review of the Literature with Meta-Analysis
Source: J Clin Med. 2022 Feb 27;11(5):1319. doi: 10.3390/jcm11051319 (PMC8910933; doi:10.3390/jcm11051319)
Supplement: Supplementary file 1 [file jcm-11-01319-s001.zip › jcm-1605693-supplementary.pdf]

Study	Women Chest pain	Women Total	Men Chest pain	Men Total	RR	95%-CI
Abdulla Shebab 2020	1681	2033	12174	13499	0.92	[0.90; 0.94]
Michele M Pelter 2012	143	198	266	367	1.00	[0.90; 1.11]
John G. Canto 2012	279425	481581	458677	661932	0.84	[0.83; 0.84]
Binquan You 2018	91	117	193	220	0.89	[0.80; 0.99]
Anoop S V Shah 2015	405	504	531	622	0.94	[0.89; 0.99]
Amy V Ferry 2019	698	756	1081	1185	1.01	[0.99; 1.04]
Nils Arne Sörensen 2018	781	880	1492	1640	0.98	[0.95; 1.00]
Holli A DeVon 2014	174	272	159	164	0.66	[0.60; 0.72]
Judith H Lichtman 2018	1748	2009	874	976	0.97	[0.95; 1.00]
Amisha Patel 2015	4921	5825	17116	19923	0.98	[0.97; 1.00]
Michael G Nanna 2019	980	1346	1326	1695	0.93	[0.89; 0.97]
Juan Ken Lee 2019	2880	4991	3569	5369	0.87	[0.84; 0.89]
Louise Hougesen Bjerking 2016	189	249	203	250	0.93	[0.85; 1.02]
**Random effects model**		**500761**		**707842**	**0.91**	**[0.86; 0.97]**

Heterogeneity: $I^2 = 99\%$, $\tau^2 = 0.0104$, $p < 0.01$

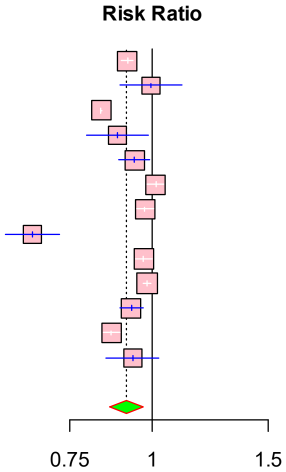

Figure S1 : chest pain

Study	Women Chest pain	Women Total	Men Chest pain	Men Total	RR	95%-CI
Abdulla Shebab 2020	1681	2033	12174	13499	0.92	[0.90; 0.94]
Michele M Pelter 2012	83	110	172	221	0.97	[0.85; 1.10]
John G. Canto 2012	279425	481581	458677	661932	0.84	[0.83; 0.84]
Binquan You 2018	91	117	193	220	0.89	[0.80; 0.99]
Anoop S V Shah 2015	96	117	130	153	0.97	[0.87; 1.08]
Amy V Ferry 2019	84	90	171	184	1.00	[0.94; 1.08]
Holli A DeVon 2014	51	77	15	24	1.06	[0.75; 1.50]
Judith H Lichtman 2018	1748	2009	874	976	0.97	[0.95; 1.00]
Amisha Patel 2015	4921	5825	17116	19923	0.98	[0.97; 1.00]
Michael G Nanna 2019	980	1346	1326	1695	0.93	[0.89; 0.97]
**Random effects model**		**493305**		**698827**	**0.94**	**[0.88; 1.01]**

Heterogeneity: $I^2 = 99\%$, $\tau^2 = 0.0104$, $p < 0.01$

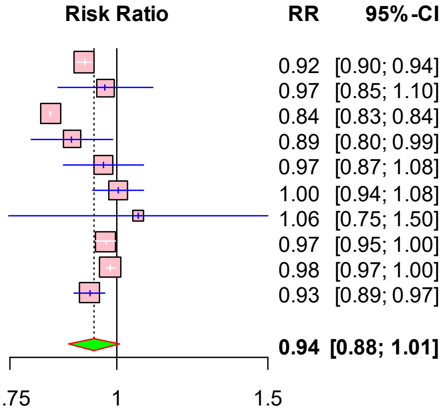

Figure S2 : Chest pain in the ACS confirmed subgroup

Study	Women Dyspnea	Women Total	Men Dyspnea	Men Total	RR	95%-CI
Michele M Pelter 2012	30	110	51	221	1.18	[0.80; 1.74]
Holli A DeVon 2014	45	77	10	24	1.40	[0.84; 2.33]
Judith H Lichtman 2018	1061	2009	465	976	1.11	[1.03; 1.20]
Amisha Patel 2015	701	5825	2142	19923	1.12	[1.03; 1.21]
Michael G Nanna 2019	656	1346	792	1695	1.04	[0.97; 1.12]
**Fixed effect model**		**9367**		**22839**	**1.10**	**[1.05; 1.15]**

Heterogeneity: $I^2 = 0\%$, $\tau^2 = 0$, $p = 0.54$

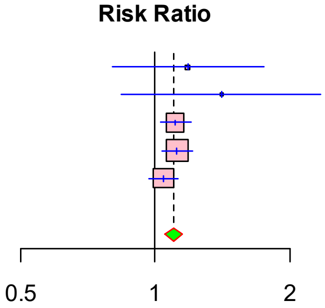

Figure S3 : Dyspnea in ACS confirmed subgroup

Study	Women Dyspnea	Women Total	Men Dyspnea	Men Total	RR	95%-CI
Michele M Pelter 2012	30	110	51	221	1.18	[0.80; 1.74]
Holli A DeVon 2014	45	77	10	24	1.40	[0.84; 2.33]
Judith H Lichtman 2018	1061	2009	465	976	1.11	[1.03; 1.20]
Amisha Patel 2015	701	5825	2142	19923	1.12	[1.03; 1.21]
Michael G Nanna 2019	656	1346	792	1695	1.04	[0.97; 1.12]
**Fixed effect model**		**9367**		**22839**	**1.10**	**[1.05; 1.15]**

Heterogeneity: $I^2 = 0\%$, $\tau^2 = 0$, $p = 0.54$

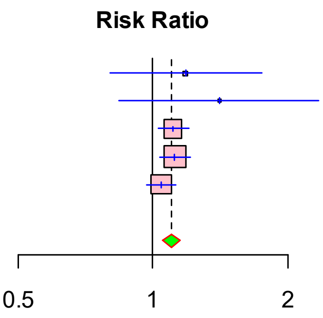

Figure S4 : Dyspnea in ACS confirmed subgroup

Study	Women Arm pain	Women Total	Men Arm pain	Men Total	RR	95%-CI
Michele M Pelter 2012	39	198	55	367	1.31	[0.91; 1.91]
Holli A DeVon 2014	98	272	74	264	1.29	[1.00; 1.65]
**Fixed effect model**		**470**		**631**	**1.30**	**[1.05; 1.59]**

Heterogeneity: $I^2 = 0\%$, $\tau^2 = 0$, $p = 0.92$

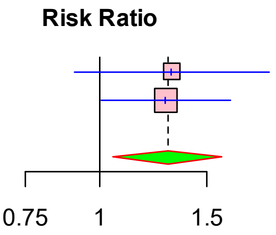

Figure S5 : arm pain

Study	Women Arm pain	Women Total	Men Arm pain	Men Total	RR	95%-CI
Michele M Pelter 2012	28	110	41	221	1.37	[0.90; 2.09]
Holli A DeVon 2014	36	77	7	24	1.60	[0.82; 3.12]
**Fixed effect model**		**187**		**245**	**1.44**	**[1.01; 2.05]**

Heterogeneity: $I^2 = 0\%$, $\tau^2 = 0$, $p = 0.70$

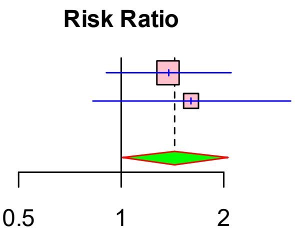

Figure S6 : Arm pain in the ACS confirmed subgroup

Study	Women Sweat	Women Total	Men Sweat	Men Total	RR	95%-CI
Michele M Pelter 2012	20	198	49	367	0.76	[0.46; 1.24]
Holli A DeVon 2014	88	272	80	264	1.07	[0.83; 1.37]
Judith H Lichtman 2018	1071	2009	542	976	0.96	[0.90; 1.03]
Nils Arne Sörensen 2018	136	880	293	1640	0.87	[0.72; 1.04]
Manon G van der Meer 2015	311	1003	420	1328	0.98	[0.87; 1.11]
Louise Hougesen Bjerking 2016	30	249	36	250	0.84	[0.53; 1.31]
**Fixed effect model**		**4611**		**4825**	**0.95**	**[0.90; 1.01]**

Heterogeneity: $I^2 = 0\%$, $\tau^2 = 0$, $p = 0.66$

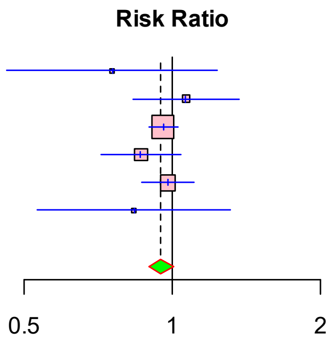

Figure S7 : Clamy skin

Study	Women Sweat	Women Total	Men Sweat	Men Total	RR	95%-CI
Michele M Pelter 2012	11	110	24	221	0.92	[0.47; 1.81]
Holli A DeVon 2014	26	77	8	24	1.01	[0.53; 1.93]
Judith H Lichtman 2018	1071	2009	542	976	0.96	[0.90; 1.03]
**Fixed effect model**		**2196**		**1221**	**0.96**	**[0.90; 1.03]**

Heterogeneity: $I^2 = 0\%$, $\tau^2 = 0$, $p = 0.98$

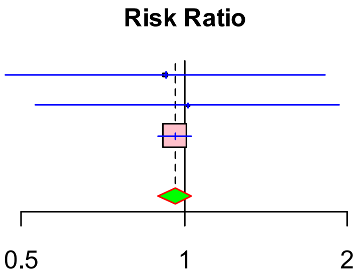

Figure S8 : Clamy skin in the ACS confirmed subgroup

Study	Women Nausea/Vomiting	Women Total	Men Nausea/Vomiting	Men Total	RR	95%-CI
Michele M Pelter 2012	32	198	44	367	1.35	[0.88; 2.05]
Holli A DeVon 2014	111	272	79	264	1.36	[1.08; 1.72]
Judith H Lichtman 2018	1236	2009	490	976	1.23	[1.14; 1.32]
Michael G Nanna 2019	597	1346	574	1695	1.31	[1.20; 1.43]
Nils Arne Sörensen 2018	228	880	255	1640	1.67	[1.42; 1.95]
Manon G van der Meer 2015	307	1003	259	1328	1.57	[1.36; 1.81]
Louise Hougesen Bjerking 2016	47	249	31	250	1.52	[1.00; 2.31]
**Random effects model**		**5957**		**6520**	**1.40**	**[1.26; 1.56]**

Heterogeneity: $I^2 = 68\%$, $\tau^2 = 0.0111$, $p < 0.01$

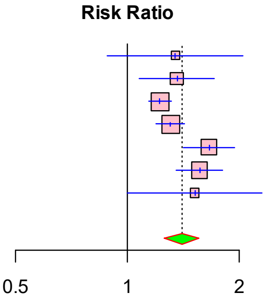

Figure S9 : nausea and vomiting

Study	Women Nausea/Vomiting	Women Total	Men Nausea/Vomiting	Men Total	RR	95%-CI
Michele M Pelter 2012	17	110	23	221	1.48	[0.83; 2.66]
Holli A DeVon 2014	29	77	7	24	1.29	[0.65; 2.57]
Judith H Lichtman 2018	1236	2009	490	976	1.23	[1.14; 1.32]
Michael G Nanna 2019	597	1346	574	1695	1.31	[1.20; 1.43]
**Fixed effect model**		**3542**		**2916**	**1.27**	**[1.20; 1.34]**

Heterogeneity: $I^2 = 0\%$, $\tau^2 = 0$, $p = 0.65$

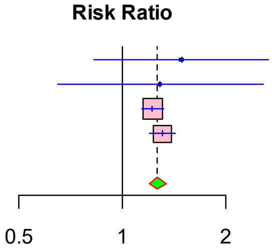

Figure S10 : Nausea-vomiting in the ACS confirmed subgroup

Study	Women Asthenia	Women Total	Men Asthenia	Men Total	RR	95%-CI
Holli A DeVon 2014	126	272	123	264	0.99	[0.83; 1.19]
Judith H Lichtman 2018	908	2009	399	976	1.11	[1.01; 1.21]
Michael G Nanna 2019	389	1346	452	1695	1.08	[0.97; 1.22]
Louise Hougesen Bjerking 2016	7	249	8	250	0.88	[0.32; 2.39]
**Fixed effect model**		**3876**		**3185**	**1.08**	**[1.01; 1.16]**

Heterogeneity: $I^2 = 0\%$, $\tau^2 = 0$, $p = 0.75$

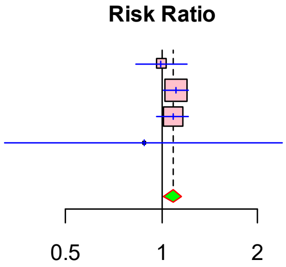

Figure S11 : weakness

Study	Women Asthenia	Women Total	Men Asthenia	Men Total	RR	95%-CI
Holli A DeVon 2014	30	77	8	24	1.17	[0.62; 2.20]
Judith H Lichtman 2018	908	2009	399	976	1.11	[1.01; 1.21]
Michael G Nanna 2019	389	1346	452	1695	1.08	[0.97; 1.22]
**Fixed effect model**		**3432**		**2695**	**1.10**	**[1.02; 1.18]**

Heterogeneity: $I^2 = 0\%$, $\tau^2 = 0$, $p = 0.95$

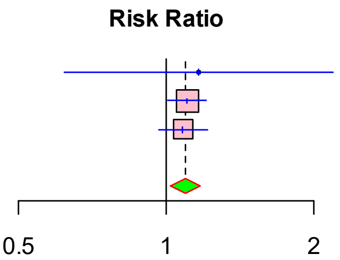

Figure S12 : weakness in the ACS confirmed subgroup

Study	Women Palpitations	Women Total	Men Palpitations	Men Total	RR	95%-CI
Michele M Pelter 2012	12	198	14	367	1.59	[0.75; 3.37]
Holli A DeVon 2014	87	272	59	264	1.43	[1.08; 1.90]
Judith H Lichtman 2018	376	2009	122	976	1.50	[1.24; 1.81]
Juan Ken Lee 2019	172	4991	106	5369	1.75	[1.37; 2.22]
Manon G van der Meer 2015	172	1003	113	1328	2.02	[1.61; 2.52]
**Fixed effect model**		**8473**		**8304**	**1.66**	**[1.49; 1.86]**

Heterogeneity: $I^2 = 24\%$, $\tau^2 = 0.0057$, $p = 0.26$

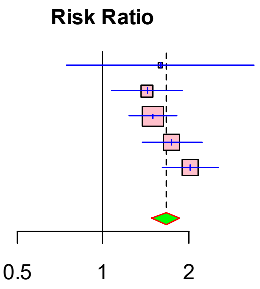

Figure S13 : palpitations

Study	Women Palpitations	Women Total	Men Palpitations	Men Total	RR	95%-CI
Michele M Pelter 2012	5	110	6	221	1.67	[0.52; 5.37]
Holli A DeVon 2014	21	77	4	24	1.64	[0.62; 4.30]
Judith H Lichtman 2018	376	2009	122	976	1.50	[1.24; 1.81]
**Fixed effect model**		**2196**		**1221**	**1.51**	**[1.25; 1.81]**

Heterogeneity: $I^2 = 0\%$, $\tau^2 = 0$, $p = 0.97$

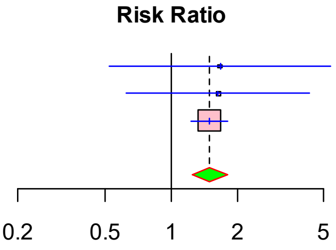

Figure S14 : palpitations in the ACS confirmed subgroup

Study	Women Shoulder pain	Women Total	Men Shoulder pain	Men Total	RR	95%-CI
Michele M Pelter 2012	14	198	15	367	1.73	[0.85; 3.51]
Holli A DeVon 2014	100	272	76	264	1.28	[1.00; 1.63]
Maria Rubini Gimenez 2014	39	796	104	1679	0.79	[0.55; 1.13]
**Random effects model**		**1266**		**2310**	**1.14**	**[0.76; 1.70]**

Heterogeneity: $I^2 = 68\%$, $\tau^2 = 0.0823$, $p = 0.04$

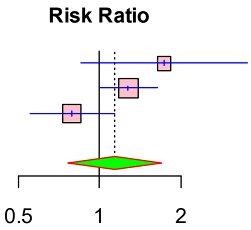

Figure S15 : shoulder pain

Study	Women Shoulder pain	Women Total	Men Shoulder pain	Men Total	RR	95%-CI
Michele M Pelter 2012	9	110	10	221	1.81	[0.76; 4.32]
Holli A DeVon 2014	34	77	6	24	1.77	[0.85; 3.69]
**Fixed effect model**		**187**		**245**	**1.78**	**[1.02; 3.13]**

Heterogeneity: $I^2 = 0\%$, $\tau^2 = 0$, $p = 0.97$

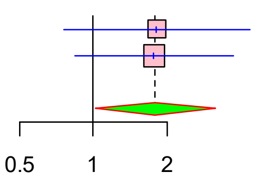

Figure S16 : Shoulder pain in the ACS confirmed subgroup

Study	Women Abdominal pain	Women Total	Men Abdominal pain	Men Total	RR	95%-CI
Maria Rubini Gimenez 2014	52	796	145	1679	0.76	[0.56; 1.03]
Louise Hougesen Bjerking 2016	14	249	8	250	1.76	[0.75; 4.11]
**Fixed effect model**		**1045**		**1929**	**0.84**	**[0.63; 1.11]**

Heterogeneity: $I^2 = 70\%$, $\tau^2 = 0.2488$, $p = 0.07$

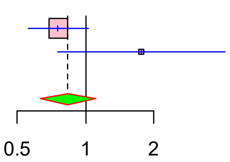

Figure S17 : Upper abdominal pain

Study	Women Total	Women Mean	Women SD	Men Total	Men Mean	Men SD	MD	95%-CI
Abdulla Shebab 2020	2033	61.73	12.6000	13499	53.27	11.9600	8.46	[7.88; 9.04]
Binquan You 2018	117	73.40	9.6000	220	71.70	8.6000	1.70	[-0.38; 3.78]
Anoop S V Shah 2015	621	70.13	15.6500	775	63.79	14.8800	6.34	[4.72; 7.96]
Amy V Ferry 2019	756	62.80	14.0000	1185	60.70	13.3000	2.10	[0.85; 3.35]
Nils Arne Sörensen 2018	880	67.00	10.0000	1640	61.00	10.5000	6.00	[5.17; 6.83]
Holli A DeVon 2014	272	60.80	15.2000	464	59.10	13.5000	1.70	[-0.48; 3.88]
Judith H Lichtman 2018	2009	47.20	6.3000	976	47.10	5.9000	0.10	[-0.36; 0.56]
Amisha Patel 2015	5825	64.40	11.7000	19923	59.30	11.9000	5.10	[4.76; 5.44]
Michael G Nanna 2019	1346	82.13	5.2200	1695	81.13	4.8400	1.00	[0.64; 1.36]
Maria Rubini Gimenez 2014	796	70.00	11.5000	1679	59.00	12.5000	11.00	[10.00; 12.00]
Juan Ken Lee 2019	4991	75.00	14.0000	5369	68.00	15.0000	7.00	[6.44; 7.56]
Manon G van der Meer 2015	1003	62.00	16.0000	1328	59.00	15.0000	3.00	[1.72; 4.28]
Louise Hougesen Bjerking 2016	249	74.00	9.5000	250	74.00	9.5000	0.00	[-1.67; 1.67]
**Random effects model**	**20898**			**49003**			**4.15**	**[2.28; 6.03]**

Heterogeneity: $I^2 = 99\%$, $\tau^2 = 11.4828$, $p < 0.01$

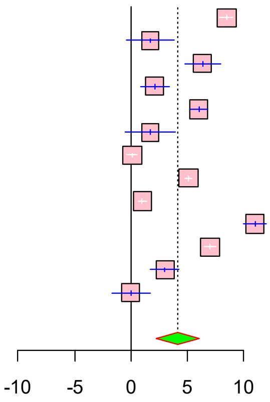

Figure S18 : age at inclusion
